# Supplementary material for: Pyruvate Dehydrogenase Kinase 1 inhibition mediated oxidative phosphorylation enhancement in cartilage promotes osteoarthritis progression
Source: BMC Musculoskelet Disord. 2023 Jul 20;24:597. doi: 10.1186/s12891-023-06585-6 (PMC10357736; doi:10.1186/s12891-023-06585-6)
Supplement: Supplementary file 1 — Additional file 1. [file 12891_2023_6585_MOESM1_ESM.zip › MF.Gsea.1653627316242/GOMF_HYDROLASE_ACTIVITY_HYDROLYZING_O_GLYCOSYL_COMPOUNDS.html]

Details for gene set GOMF\_HYDROLASE\_ACTIVITY\_HYDROLYZING\_O\_GLYCOSYL\_COMPOUNDS[GSEA]

|  || Dataset | 55457\_collapsed\_to\_symbols.cls.cls #OA\_versus\_normal.cls.cls #OA\_versus\_normal\_repos |
| Phenotype | cls.cls#OA\_versus\_normal\_repos |
| Upregulated in class | OA |
| GeneSet | GOMF\_HYDROLASE\_ACTIVITY\_HYDROLYZING\_O\_GLYCOSYL\_COMPOUNDS |
| Enrichment Score (ES) | 0.2998208 |
| Normalized Enrichment Score (NES) | 1.2849555 |
| Nominal p-value | 0.08585858 |
| FDR q-value | 0.4140685 |
| FWER p-Value | 1.0 |
Table: GSEA Results Summary

  

Fig 1: Enrichment plot: GOMF\_HYDROLASE\_ACTIVITY\_HYDROLYZING\_O\_GLYCOSYL\_COMPOUNDS      
 Profile of the Running ES Score & Positions of GeneSet Members on the Rank Ordered List

  

| SYMBOL | TITLE | RANK IN GENE LIST | RANK METRIC SCORE | RUNNING ES | CORE ENRICHMENT || 1 | SPAM1 | sperm adhesion molecule 1 [Source:HGNC Symbol;Acc:HGNC:11217] | 180 | 0.831 | 0.0246 | Yes |
| 2 | GLB1 | galactosidase beta 1 [Source:HGNC Symbol;Acc:HGNC:4298] | 337 | 0.731 | 0.0465 | Yes |
| 3 | ABHD10 | "abhydrolase domain containing 10, depalmitoylase [Source:HGNC Symbol;Acc:HGNC:25656]" | 437 | 0.690 | 0.0709 | Yes |
| 4 | GALC | galactosylceramidase [Source:HGNC Symbol;Acc:HGNC:4115] | 478 | 0.669 | 0.0988 | Yes |
| 5 | GUSB | glucuronidase beta [Source:HGNC Symbol;Acc:HGNC:4696] | 904 | 0.541 | 0.0909 | Yes |
| 6 | MANEA | mannosidase endo-alpha [Source:HGNC Symbol;Acc:HGNC:21072] | 950 | 0.528 | 0.1120 | Yes |
| 7 | HYAL1 | hyaluronidase 1 [Source:HGNC Symbol;Acc:HGNC:5320] | 997 | 0.518 | 0.1325 | Yes |
| 8 | MAN2A1 | mannosidase alpha class 2A member 1 [Source:HGNC Symbol;Acc:HGNC:6824] | 1069 | 0.501 | 0.1503 | Yes |
| 9 | GAA | alpha glucosidase [Source:HGNC Symbol;Acc:HGNC:4065] | 1149 | 0.486 | 0.1667 | Yes |
| 10 | GANAB | glucosidase II alpha subunit [Source:HGNC Symbol;Acc:HGNC:4138] | 1267 | 0.464 | 0.1792 | Yes |
| 11 | MAN1C1 | mannosidase alpha class 1C member 1 [Source:HGNC Symbol;Acc:HGNC:19080] | 1275 | 0.463 | 0.2002 | Yes |
| 12 | CTBS | chitobiase [Source:HGNC Symbol;Acc:HGNC:2496] | 1333 | 0.453 | 0.2168 | Yes |
| 13 | HEXB | hexosaminidase subunit beta [Source:HGNC Symbol;Acc:HGNC:4879] | 1434 | 0.438 | 0.2294 | Yes |
| 14 | FUCA1 | alpha-L-fucosidase 1 [Source:HGNC Symbol;Acc:HGNC:4006] | 1506 | 0.428 | 0.2438 | Yes |
| 15 | GLB1L | galactosidase beta 1 like [Source:HGNC Symbol;Acc:HGNC:28129] | 1544 | 0.421 | 0.2605 | Yes |
| 16 | NAGPA | N-acetylglucosamine-1-phosphodiester alpha-N-acetylglucosaminidase [Source:HGNC Symbol;Acc:HGNC:17378] | 1608 | 0.412 | 0.2748 | Yes |
| 17 | NAGA | alpha-N-acetylgalactosaminidase [Source:HGNC Symbol;Acc:HGNC:7631] | 1674 | 0.402 | 0.2884 | Yes |
| 18 | GBA3 | glucosylceramidase beta 3 (gene/pseudogene) [Source:HGNC Symbol;Acc:HGNC:19069] | 1767 | 0.388 | 0.2993 | Yes |
| 19 | EDEM2 | ER degradation enhancing alpha-mannosidase like protein 2 [Source:HGNC Symbol;Acc:HGNC:15877] | 2076 | 0.353 | 0.2917 | Yes |
| 20 | GM2A | GM2 ganglioside activator [Source:HGNC Symbol;Acc:HGNC:4367] | 2299 | 0.327 | 0.2896 | Yes |
| 21 | HPSE | heparanase [Source:HGNC Symbol;Acc:HGNC:5164] | 2360 | 0.319 | 0.2998 | Yes |
| 22 | NAGLU | N-acetyl-alpha-glucosaminidase [Source:HGNC Symbol;Acc:HGNC:7632] | 2767 | 0.273 | 0.2809 | No |
| 23 | HEXA | hexosaminidase subunit alpha [Source:HGNC Symbol;Acc:HGNC:4878] | 3025 | 0.247 | 0.2724 | No |
| 24 | HPSE2 | heparanase 2 (inactive) [Source:HGNC Symbol;Acc:HGNC:18374] | 3318 | 0.219 | 0.2598 | No |
| 25 | GBA | glucosylceramidase beta [Source:HGNC Symbol;Acc:HGNC:4177] | 3327 | 0.219 | 0.2694 | No |
| 26 | MAN1B1 | mannosidase alpha class 1B member 1 [Source:HGNC Symbol;Acc:HGNC:6823] | 3447 | 0.207 | 0.2697 | No |
| 27 | AGL | "amylo-alpha-1, 6-glucosidase, 4-alpha-glucanotransferase [Source:HGNC Symbol;Acc:HGNC:321]" | 3537 | 0.199 | 0.2721 | No |
| 28 | MAN2B1 | mannosidase alpha class 2B member 1 [Source:HGNC Symbol;Acc:HGNC:6826] | 3577 | 0.196 | 0.2781 | No |
| 29 | CEMIP | cell migration inducing hyaluronidase 1 [Source:HGNC Symbol;Acc:HGNC:29213] | 3747 | 0.182 | 0.2734 | No |
| 30 | CHI3L2 | chitinase 3 like 2 [Source:HGNC Symbol;Acc:HGNC:1933] | 3821 | 0.174 | 0.2758 | No |
| 31 | SI | sucrase-isomaltase [Source:HGNC Symbol;Acc:HGNC:10856] | 4021 | 0.154 | 0.2675 | No |
| 32 | GLA | galactosidase alpha [Source:HGNC Symbol;Acc:HGNC:4296] | 4172 | 0.140 | 0.2623 | No |
| 33 | MAN1A2 | mannosidase alpha class 1A member 2 [Source:HGNC Symbol;Acc:HGNC:6822] | 4272 | 0.131 | 0.2607 | No |
| 34 | NEU1 | neuraminidase 1 [Source:HGNC Symbol;Acc:HGNC:7758] | 4486 | 0.114 | 0.2494 | No |
| 35 | MAN2B2 | mannosidase alpha class 2B member 2 [Source:HGNC Symbol;Acc:HGNC:29623] | 4548 | 0.109 | 0.2497 | No |
| 36 | KL | klotho [Source:HGNC Symbol;Acc:HGNC:6344] | 4901 | 0.080 | 0.2260 | No |
| 37 | LYZL6 | lysozyme like 6 [Source:HGNC Symbol;Acc:HGNC:29614] | 4929 | 0.078 | 0.2275 | No |
| 38 | IDUA | alpha-L-iduronidase [Source:HGNC Symbol;Acc:HGNC:5391] | 5002 | 0.073 | 0.2253 | No |
| 39 | CHI3L1 | chitinase 3 like 1 [Source:HGNC Symbol;Acc:HGNC:1932] | 5598 | 0.024 | 0.1801 | No |
| 40 | EDEM1 | ER degradation enhancing alpha-mannosidase like protein 1 [Source:HGNC Symbol;Acc:HGNC:18967] | 5633 | 0.021 | 0.1784 | No |
| 41 | NEU2 | neuraminidase 2 [Source:HGNC Symbol;Acc:HGNC:7759] | 5775 | 0.010 | 0.1679 | No |
| 42 | EDEM3 | ER degradation enhancing alpha-mannosidase like protein 3 [Source:HGNC Symbol;Acc:HGNC:16787] | 6073 | -0.010 | 0.1452 | No |
| 43 | MANBA | mannosidase beta [Source:HGNC Symbol;Acc:HGNC:6831] | 6373 | -0.033 | 0.1235 | No |
| 44 | OGA | O-GlcNAcase [Source:HGNC Symbol;Acc:HGNC:7056] | 7086 | -0.086 | 0.0720 | No |
| 45 | GLB1L2 | galactosidase beta 1 like 2 [Source:HGNC Symbol;Acc:HGNC:25129] | 7341 | -0.105 | 0.0571 | No |
| 46 | CHIT1 | chitinase 1 [Source:HGNC Symbol;Acc:HGNC:1936] | 8207 | -0.172 | -0.0023 | No |
| 47 | HYAL4 | hyaluronidase 4 [Source:HGNC Symbol;Acc:HGNC:5323] | 8209 | -0.172 | 0.0057 | No |
| 48 | LCT | lactase [Source:HGNC Symbol;Acc:HGNC:6530] | 8444 | -0.189 | -0.0038 | No |
| 49 | MAN2C1 | mannosidase alpha class 2C member 1 [Source:HGNC Symbol;Acc:HGNC:6827] | 9342 | -0.259 | -0.0616 | No |
| 50 | NEU3 | neuraminidase 3 [Source:HGNC Symbol;Acc:HGNC:7760] | 9348 | -0.259 | -0.0499 | No |
| 51 | CHIA | chitinase acidic [Source:HGNC Symbol;Acc:HGNC:17432] | 9641 | -0.287 | -0.0593 | No |
| 52 | TREH | trehalase [Source:HGNC Symbol;Acc:HGNC:12266] | 9910 | -0.313 | -0.0657 | No |
| 53 | MGAM | maltase-glucoamylase [Source:HGNC Symbol;Acc:HGNC:7043] | 10106 | -0.331 | -0.0655 | No |
| 54 | MAN1A1 | mannosidase alpha class 1A member 1 [Source:HGNC Symbol;Acc:HGNC:6821] | 10207 | -0.341 | -0.0574 | No |
| 55 | GBE1 | "1,4-alpha-glucan branching enzyme 1 [Source:HGNC Symbol;Acc:HGNC:4180]" | 10356 | -0.357 | -0.0523 | No |
| 56 | HYAL2 | hyaluronidase 2 [Source:HGNC Symbol;Acc:HGNC:5321] | 10420 | -0.363 | -0.0403 | No |
| 57 | LYZ | lysozyme [Source:HGNC Symbol;Acc:HGNC:6740] | 10483 | -0.371 | -0.0279 | No |
| 58 | MOGS | mannosyl-oligosaccharide glucosidase [Source:HGNC Symbol;Acc:HGNC:24862] | 10630 | -0.386 | -0.0213 | No |
| 59 | OVGP1 | oviductal glycoprotein 1 [Source:HGNC Symbol;Acc:HGNC:8524] | 10740 | -0.398 | -0.0113 | No |
| 60 | PGGHG | protein-glucosylgalactosylhydroxylysine glucosidase [Source:HGNC Symbol;Acc:HGNC:26210] | 11033 | -0.434 | -0.0138 | No |
| 61 | ENGASE | endo-beta-N-acetylglucosaminidase [Source:HGNC Symbol;Acc:HGNC:24622] | 11056 | -0.439 | 0.0048 | No |
| 62 | AMY1A | amylase alpha 1A [Source:HGNC Symbol;Acc:HGNC:474] | 11681 | -0.529 | -0.0192 | No |
| 63 | MAN2A2 | mannosidase alpha class 2A member 2 [Source:HGNC Symbol;Acc:HGNC:6825] | 11772 | -0.545 | -0.0008 | No |
| 64 | PARG | poly(ADP-ribose) glycohydrolase [Source:HGNC Symbol;Acc:HGNC:8605] | 11786 | -0.547 | 0.0236 | No |
| 65 | CEMIP2 | cell migration inducing hyaluronidase 2 [Source:HGNC Symbol;Acc:HGNC:11869] | 11971 | -0.588 | 0.0366 | No |
| 66 | GNE | glucosamine (UDP-N-acetyl)-2-epimerase/N-acetylmannosamine kinase [Source:HGNC Symbol;Acc:HGNC:23657] | 12538 | -0.767 | 0.0282 | No |
Table: GSEA details [plain text format]

  

Fig 2: GOMF\_HYDROLASE\_ACTIVITY\_HYDROLYZING\_O\_GLYCOSYL\_COMPOUNDS      
 Blue-Pink O' Gram in the Space of the Analyzed GeneSet

  

Fig 3: GOMF\_HYDROLASE\_ACTIVITY\_HYDROLYZING\_O\_GLYCOSYL\_COMPOUNDS: Random ES distribution      
 Gene set null distribution of ES for **GOMF\_HYDROLASE\_ACTIVITY\_HYDROLYZING\_O\_GLYCOSYL\_COMPOUNDS**
